# A novel naphthalimide derivative reduces platelet activation and thrombus formation via suppressing GPVI

**DOI:** 10.1111/jcmm.16886

**Published:** 2021-08-27

**Authors:** Tzenge‐Lien Shih, Kuan‐Hung Lin, Ray‐Jade Chen, Ting‐Yu Chen, Wei‐Ting Kao, Jen‐Wei Liu, Hsueh‐Hsiao Wang, Hsien‐Yu Peng, Yu‐Yo Sun, Wan‐Jung Lu

**Affiliations:** ^1^ Department of Chemistry Tamkang University New Taipei City Taiwan; ^2^ Traditional Herbal Medicine Research Center Taipei Medical University Hospital Taipei Taiwan; ^3^ Department of Pharmacology School of Medicine College of Medicine Taipei Medical University Taipei Taiwan; ^4^ Institute of Biomedical Sciences MacKay Medical College New Taipei City Taiwan; ^5^ Division of General Surgery Department of Surgery Taipei Medical University Hospital Taipei Taiwan; ^6^ Department of Surgery School of Medicine College of Medicine Taipei Medical University Taipei Taiwan; ^7^ Department of Medicine Mackay Medical College New Taipei City Taiwan; ^8^ Department of Neuroscience Center for Brain Immunology and Glia (BIG) University of Virginia School of Medicine Charlottesville VA USA; ^9^ Department of Medical Research Taipei Medical University Hospital Taipei Taiwan; ^10^ Graduate Institute of Metabolism and Obesity Sciences College of Nutrition Taipei Medical University Taipei Taiwan

**Keywords:** antiplatelet activity, bleeding risk, collagen, naphthalimide derivatives, platelet activation, thrombus formation

## Abstract

Naphthalimide derivatives have multiple biological activities, including antitumour and anti‐inflammatory activities. We previously synthesized several naphthalimide derivatives; of them, compound **5** was found to exert the strongest inhibitory effect on human DNA topoisomerase II activity. However, the effects of naphthalimide derivatives on platelet activation have not yet been investigated. Therefore, the mechanism underlying the antiplatelet activity of compound **5** was determined in this study. The data revealed that compound **5** (5–10 μM) inhibited collagen‐ and convulxin‐ but not thrombin‐ or U46619‐mediated platelet aggregation, suggesting that compound **5** is more sensitive to the inhibition of glycoprotein VI (GPVI) signalling. Indeed, compound **5** could inhibit the phosphorylation of signalling molecules downstream of GPVI, followed by the inhibition of calcium mobilization, granule release and GPIIb/IIIa activation. Moreover, compound **5** prevented pulmonary embolism and prolonged the occlusion time, but tended to prolong the bleeding time, indicating that it can prevent thrombus formation but may increase bleeding risk. This study is the first to demonstrate that the naphthalimide derivative compound **5** exerts antiplatelet and antithrombotic effects. Future studies should modify compound **5** to synthesize more potent and efficient antiplatelet agents while minimizing bleeding risk, which may offer a therapeutic potential for cardiovascular diseases.

## 1 INTRODUCTION

Normal haemostasis is a complicated process involving platelets and the coagulation cascade.^1^ When blood vessels are injured, circulating platelets adhere to exposed extracellular matrix proteins, such as von Willebrand factor and collagen. The exposed collagen can activate platelets through glycoprotein VI (GPVI). GPVI, a collagen receptor present on platelets, is associated with an immunoreceptor tyrosine‐based activation motif (ITAM) containing the dimeric Fc receptor γ‐chain. The binding of collagen to GPVI can activate Src family kinases (SFKs) Fyn and Lyn and subsequently induce ITAM tyrosine phosphorylation, thus resulting in the recruitment and activation of the tyrosine kinase Syk.^2^, ^3^ A signalling complex in turn forms and conducts signalling cascade that activates phospholipase Cγ2 (PLCγ2), and its downstream effectors including protein kinase C (PKC) and mitogen‐activated protein kinase (MAPKs), such as p38 MAPK, extracellular‐signal‐regulated kinase (ERK) and c‐Jun amino‐terminal kinase (JNK),^2^, ^3^, ^4^ promoting the release of ADP and thromboxane A_2_ that can further amplify platelet activation and recruit more platelets from the bloodstream to participate in haemostasis. Additionally, collagen activates protein kinase B (Akt) downstream of GPVI, which regulates platelet secretion and calcium mobilization.^5^, ^6^ These activation cascades finally result in the formation of a firm platelet plug at the injury site to stop blood loss. Inappropriate regulation of the haemostatic process may lead to pathological consequences such as bleeding and thrombosis. Antiplatelet agents are used to prevent or treat secondary ischaemic stroke and myocardial infarction. However, the clinically significant bleeding has limited their use,^7^, ^8^ necessitating the development of new antiplatelet agents with minimal bleeding risk.

**FIGURE 1:**
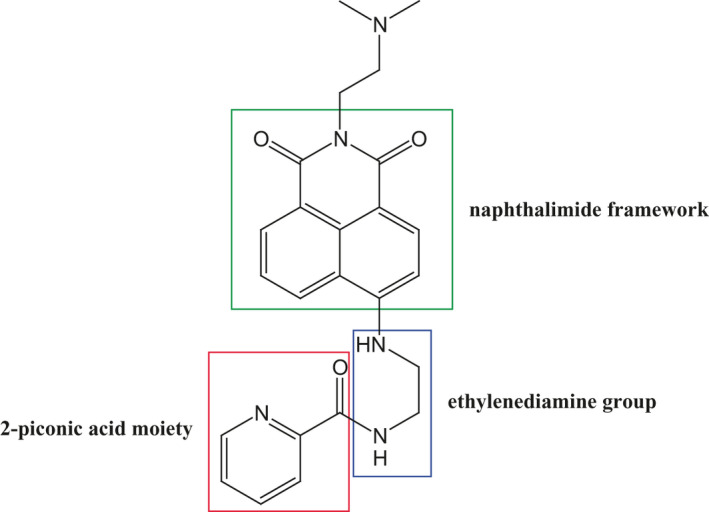
Structure of compound **5**. Compound **5** consists of a naphthalimide framework (green box) in which the C4 position is linked with an ethylenediamine group (blue box) where the amino group couples with a 2‐piconic acid moiety (red box)

Most compounds containing the naphthalimide moiety are fluorescent and have multiple biological activities including antitumuor, anti‐inflammatory and antiviral avtivities.^9^ Naphthalimide derivatives can easily intercalate into DNA and block cell division because of their flat structures.^9^, ^10^ Moreover, these derivatives can intercalate into topoisomerase II (topo II), an enzyme with DNA breakage‐reunion activity.^10^ Because of the aforementioned properties, naphthalimide derivatives can be potential antitumour agents. Moreover, many researchers have synthesized novel naphthalimide derivatives to develop more potent and efficient antitumour drugs with low toxicity.^11^, ^12^, ^13^, ^14^ Previously, we synthesized several naphthalimide derivatives^15^, ^16^ and evaluated their anticancer effect. Among these derivatives, compound **5** (Figure 1) exhibited the highest potency in inhibiting human DNA topo II activity (IC_50_ = 2.6 ± 0.1 μM) in murine B16F10 melanoma cells.^16^ In addition, **7b**, a naphthalimide derivative, could inhibit lipopolysaccharide (LPS)‐induced nuclear factor‐kappa B (NF‐κB) activation in RAW264.7 macrophages.^17^ Moreover, naphthalimides antagonize NS1 and influenza virus and thus inhibit viral replication; this process might be mediated by REDD1.^18^ Although these naphthalimide derivatives have been reported to be involved in several pathophysiological processes, their effects on platelet activation have not been investigated. Thus, in the present study, we examined the antiplatelet mechanism of compound **5**. We expect that compound **5** be a lead compound for the development of novel antiplatelet agents for treating cardiovascular diseases.

## 2 MATERIALS AND METHODS

### 2.1 Materials

Thrombin, U46619, collagen and convulxin were purchased from Chrono‐Log. Fluorescein sodium, phorbol 12,13‐dibutyrate (PDBu), luciferase/luciferin and ADP were purchased from Sigma. Fura 2‐AM; anti‐phospho‐Lyn (Tyr^507^), anti‐phospho‐Akt (Ser^473^), anti‐phospho‐Fyn (Tyr^530^) and anti‐Lyn polyclonal antibodies (pAbs); and anti‐Fyn monoclonal antibodies (mAbs) were purchased from Abcam. Anti‐pleckstrin (p47) and anti‐phospho‐ERK1 (Thr^202^/Tyr^204^)/ERK2 (Thr^185^/Tyr^187^) pAbs were purchased from GeneTex. Anti‐phospho‐(Ser) PKC substrate, anti‐phospho‐JNK (Thr^183^/Tyr^185^), anti‐phospho‐p38 MAPK (Thr^180^/Tyr^182^) and anti‐JNK pAbs; and anti‐Akt, anti‐p38 MAPK and anti‐ERK mAbs were purchased from Cell Signaling. Horseradish peroxidase (HRP)‐conjugated AffiniPure goat antirabbit, AffiniPure goat antimouse and AffiniPure donkey antigoat immunoglobulin G (IgG) were purchased from Jackson ImmunoResearch. Allophycocyanin (APC)‐conjugated PAC‐1 antibodies and anti‐P‐selectin were purchased from Biolegend. Hybond‐P polyvinylidene difluoride (PVDF) membrane was purchased from GE Healthcare Life Sciences. A SuperLight Chemiluminescent HRP kit was purchased from Bionovas.

### 2.2 Synthesis of compound 5

Compound **5** was synthesized as described previously.^16^ Compound **5** consists of a naphthalimide framework in which the C4 position is linked with an ethylenediamine group where the amino group couples with a 2‐piconic acid moiety (Figure 1). Compound **5** was dissolved in DMSO and stored at 4°C until use.

### 2.3 Preparation of platelet suspensions

This study was approved by the Taipei Medical University—Joint Institutional Review Board (TMU‐JIRB‐No. N202003148) and conformed to principles outlined in the Declaration of Helsinki. All volunteers provided informed consent prior to participation in this study. Washed human platelets were prepared as described previously.^19^ In brief, whole blood was obtained from healthy participants who had not received any medicines such as nonsteroidal anti‐inflammatory drugs and aspirin during the preceding 2 weeks. Whole blood was drawn into polypropylene plastic tubes filled with an acid citrate/dextrose solution (A.C.D; 9:1, v/v). After mixing the blood samples at 120*g* for 10 min, the platelet‐rich plasma (PRP; upper layer) was collected and supplemented with prostaglandin E_1_ and heparin. After further centrifugation at 500 *g* for 10 min, platelet pellets were washed twice. The washed platelets were resuspended in Tyrode's solution supplemented with 3.5 mg/ml bovine serum albumin (BSA) to obtain platelet suspensions. The final Ca^2+^ concentration in platelet suspensions (3.6 × 10^8^ cells/ml) was 1 mM.

### 2.4 Platelet aggregation

Platelet aggregation was measured using a lumi‐aggregometer (Payton) according to the turbidimetric method.^19^ In brief, platelet suspensions (3.6 × 10^8^ cells/ml) were treated with compound **5** (1–100 μM) or an isovolumetric solvent control (0.1% DMSO) for 3 min. Subsequently, various agonists, namely collagen, thrombin and U46619, were added. Platelet aggregation was recorded for an additional 6 min.

### 2.5 Western blotting

Western blotting was performed as described previously.^20^ In brief, platelet suspensions (3.6 × 10^8^ cells/ml) were pretreated with compound **5** (5 and 10 μM) or 0.1% DMSO for 3 min and then treated again with collagen for 6 min. After centrifugation, platelet pellets were immediately resuspended in lysis buffer (200 μl) for 1 h. The supernatants were collected after centrifugation at 5000 *g* for 5 min. The protein extracts (80 μg) were subjected to 8%–12% sodium dodecylsulfate‐polyacrylamide gel electrophoresis. Separated proteins were then electrotransferred onto the polyvinylidene fluoride (PVDF) membrane through a semidry transfer (Thermo Fisher). The membrane was blocked with TBST (10 mM Tris‐base, 100 mM NaCl and 0.01% Tween 20) containing 5% BSA for 1 h. After being washed three times, the membranes were incubated with various specific primary antibodies (1:1000). Subsequently, the membrane was incubated with HRP‐conjugated antigoat, antimouse or antirabbit IgG (1:5000) for 1 h. Immunoreactive bands were developed using an electrochemiluminescence kit and analysed using Celvin S (Biostep).

### 2.6 ATP release and calcium mobilization

This method was performed as described previously.^21^ In brief, luciferase/luciferin and Fura 2‐AM were used to detect ATP release and calcium mobilization respectively. The intensity of luminescence (ATP release) and the ratio (wavelength = 340/380 nm) of fluorescence (calcium mobilization) were measured using a Hitachi Spectrometer F‐7000 in accordance with the manufacturer's instructions.

### 2.7 Flow cytometry

Flow cytometry was performed as described previously.^20^ In brief, 20 min after collagen stimulation, platelets were fixed and labelled with P‐selectin or PAC‐1 antibodies conjugated with APC for 30 min to detect the surface expression of P‐selectin and the level of GPIIb/IIIa activation respectively. After centrifugation and washing, platelets were suspended in 1 ml of phosphate‐buffered saline and measured using a CytoFLEX flow cytometer (Beckman Coulter Life Sciences). In the flow cytometry setting, platelets were gated by a forward scatter and a side scatter, and the number of events at 10,000 counts was stopped. All experiments were performed at least three times to ensure reliability.

### 2.8 Animals

Male ICR mice (weighing 20–25 g and aged 5–6 weeks) were purchased from BioLasco. This project involving the use of animals was approved by the Affidavit of Approval of Animal Use Protocol—Taipei Medical University (LAC‐2020‐0074). All animal experiments were conducted in accordance with the Guide for the Care and Use of Laboratory Animals (Eighth Edition, 2011).

### 2.9 Washed mouse platelet preparation and aggregation study

Mouse blood was collected through cardiac puncture into a tube containing 100 µl of sodium citrate followed by gentle mixing. After centrifugation at 180 *g* for 5 min, PRP was obtained and mixed with A.C.D. (9:1, v:v). The platelet pellet was obtained after centrifugation at 1300 *g* for 15 min and then resuspended in Tyrode's solution. A lumi‐aggregometer (Payton Associates) was used to measure platelet aggregation as described previously.^19^ Platelet suspensions (1.5 × 10^8^/ml) were stimulated using collagen (1 µg/ml) for 10 min; the extent of aggregation is expressed in light transmission units.

### 2.10 Pulmonary embolism in mice induced by collagen/epinephrine

Pulmonary thromboembolism was induced using collagen and epinephrine in male ICR mice according to the method described by DiMinno and Silver.^22^ In brief, mice were intravenously injected with a bolus dose of DMSO (solvent control), compound **5** (2.3 and 4.5 mg/kg) or aspirin (20 mg/kg, positive control). Mice received collagen (0.6 mg/kg) plus epinephrine (0.2 mg/kg) through tail vein injection. When the respiration is very weak and their heart was still beating, 0.5 ml of Evans blue solution (1% in saline) was injected into their heart. The lungs were excised and photographed. The mortality of mice was recorded over 24 h, and all surviving mice were sacrificed immediately after the experiment. Each group consisted of six animals. A formula for dose translation based on the body surface area was used to calculate the dose for use in the mice.^23^

### 2.11 Thrombus formation in the mesenteric microvessels of mice irradiated by fluorescein sodium

Mice were anaesthetized using 3% isoflurane with oxygen‐air mixture at a gas flow rate of 1.5–2 L/min. A bolus dose of compound **5** (1.2, 2.3 and 4.5 mg/kg), DMSO (solvent control) or aspirin (20 mg/kg, positive control) was intravenously administered through the tail vein prior to the administration of sodium fluorescein (15 mg/kg). The small intestinal segments were placed on a transparent culture dish, and the mesenteric vessels were observed under a microscope.^24^ Venules (20–30 μm) were chosen and irradiated with light (wavelength <520 nm) to induce endothelial damage‐causing thrombus formation and subsequent vessel occlusion.^24^ The time required to occlude a microvessel was recorded. A formula for dose translation based on the body surface area was used to calculate the dose for use in the mice.^23^

### 2.12 Tail‐bleeding assay

Mice were anaesthetized using 3% isoflurane with oxygen‐air mixture at a gas flow rate of 1.5–2  L/min. A bolus dose of compound **5** (1.2, 2.3 and 4.5 mg/kg), DMSO (solvent control) or aspirin (20 mg/kg, positive control) was intravenously administered for 30 min. Next, a 3‐mm incision was made from the tail tip to induce tail bleeding. The bleeding tail stump was immediately immersed in saline, and the bleeding time, which was defined as the time until no sign of bleeding was observed for at least 10 s, was recorded.^20^ A formula for dose translation based on the body surface area was used to calculate the dose for use in the mice.^23^

### 2.13 Statistical analysis

Data were analysed using analysis of variance with a post hoc analysis performed using the Newman‐Keuls test. For survival analysis, survival curves were plotted using the Kaplan‐Meier curves and analysed using the log‐rank test, and all pair‐wise multiple comparison procedures were performed by the Holm‐Sidak method. Results are expressed as the mean ± standard error of the mean (SEM). *p *< 0.05 was considered statistically significant.

## 3 RESULTS

### 3.1 Compound 5 blocks collagen‐induced platelet aggregation

**FIGURE 2:**
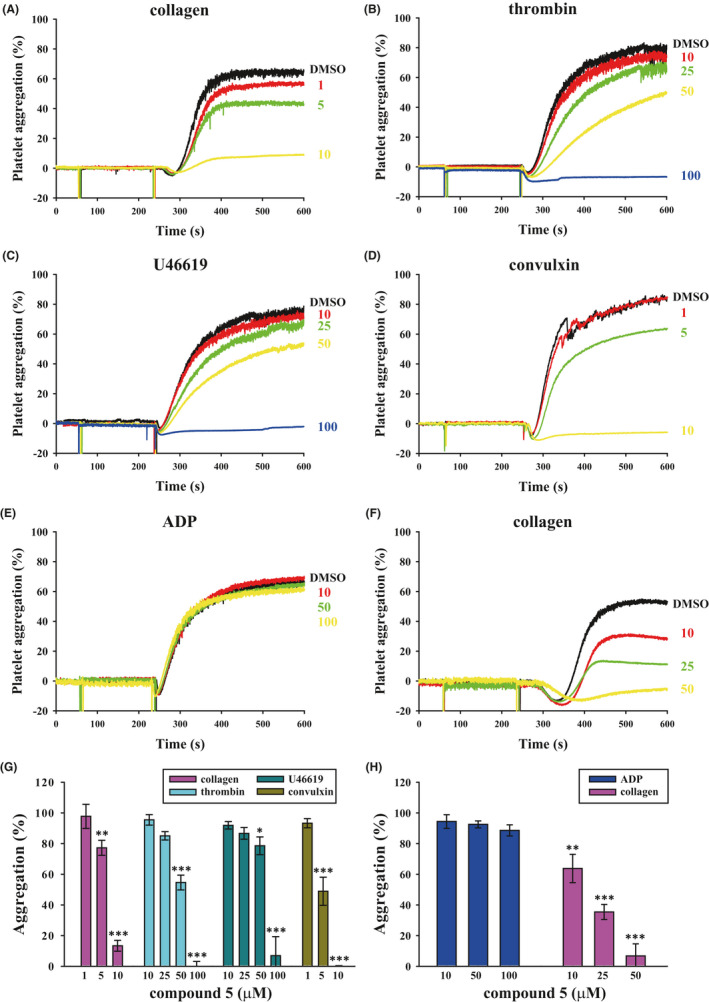
Effects of compound **5** on platelet aggregation stimulated by various agonists in human platelets. (A‐D) Washed platelets (3.6 × 10^8^ cells/ml) or (E,F) citrated PRP were treated with compound **5** (1–100 μM) or DMSO (solvent control) prior to the addition of 1 μg/ml collagen (A, F), 0.02 U/ml thrombin (B), 1 μM U46619 (C), 5 ng/ml convulxin (D) or 20 μM ADP (E). Panels A–F indicate the platelet aggregation curve, and panels G and H display the statistical analysis results for the aggregation (%) of panels (A–D) and panels (E and F) respectively. Data (D) are presented as means ± SEM (*n* = 4). *p < 0.05, **p < 0.01 and ***p < 0.001 compared with the DMSO (solvent control) group

Various platelet agonists were used to investigate the effect of compound **5** on platelet functions. As illustrated in Figure 2A, compound **5** (5–10 μM) significantly reduced collagen‐induced platelet aggregation in washed platelets. The IC_50_ was approximately 6.5 μM. Compound **5** could block thrombin‐ or U46619‐stimulated platelet aggregation (Figure 2B,C, respectively) in washed platelets only at a relatively high concentration (25–100 μM). These findings suggest that compound **5** may be more sensitive to the inhibition of collagen‐induced platelet activation. Moreover, compound **5** (5–10 μM) reduced platelet aggregation by the collagen receptor GPVI agonist convulxin (Figure 2D), confirming that compound **5** may prevent platelet activation, at least in part, through GPVI signalling. In addition, ADP‐induced platelet aggregation was conducted in PRP. As shown in Figure 2E,F, in PRP, compound **5** (10–100 μM) did not affect ADP‐induced platelet aggregation; however, compound **5** affected collagen‐induced platelet aggregation at a relatively high concentration (10–50 μM). This finding suggested that plasma protein binding may interfere with the efficacy of compound **5**. The findings of these statistical analyses are illustrated in Figure 2G (washed platelets) and 2H (PRP). Compound **5** alone did not cause platelet activation as evidenced by observing shape change (Figure S1) and did not cause platelet cytotoxicity as detected by LDH cytotoxicity assay (Figure S2). On the basis of these results, we investigated the effect of compound **5** on collagen receptor downstream signalling in subsequent experiments.

### 3.2 Compound 5 attenuates platelet activation by suppressing GPVI signalling

**FIGURE 3:**
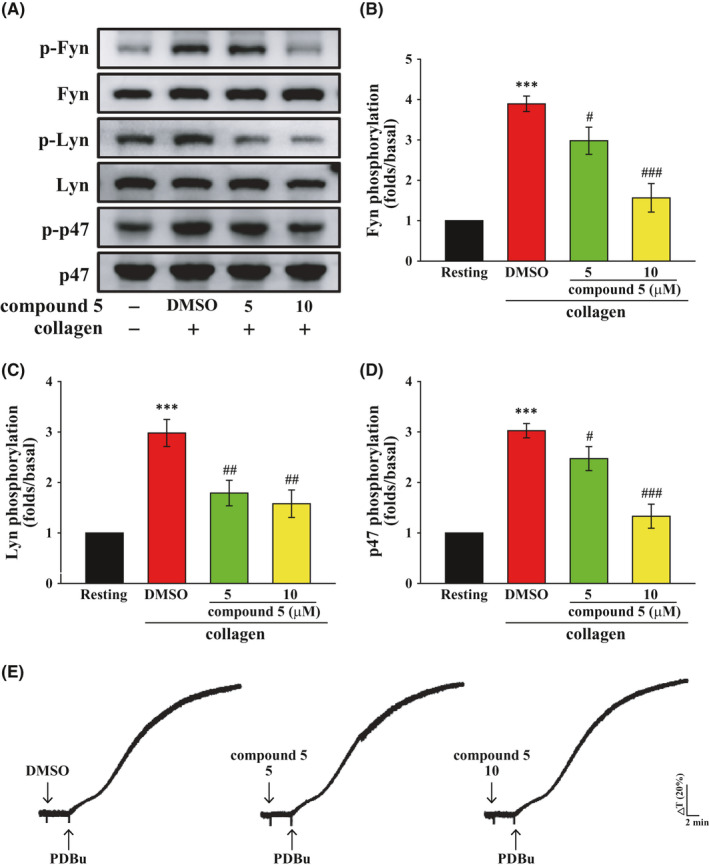
Effects of compound **5** on the collagen‐mediated phosphorylation of Fyn, Lyn and PKC substrates in human platelets. (A‐D) Washed platelets (3.6 × 10^8^ cells/ml) were treated with compound **5** (5 and 10 μM) or DMSO prior to treatment with collagen (1 μg/ml). Protein extracts of platelets were subjected to Western blotting. The total and phosphorylated Fyn, Lyn and PKC substrates (p47) were detected using specific antibodies. (E) Effects of compound **5** (5 and 10 μM) on PDBu (150 nM)‐mediated platelet aggregation. Profiles (A and E) are representative examples of three similar experiments. Data (B–D) are presented as means ± SEM (*n* = 5). ***p < 0.001 compared with the resting group. ^#^ *p *< 0.05, ^##^ *p *< 0.01 and ^###^ *p *< 0.001 compared with the DMSO (solvent control) group

The binding of collagen to GPVI can activate SFKs Fyn and Lyn and subsequently activate PLCγ2‐PKC and MAPKs.^2^, ^3^, ^4^ Thus, we examined the effect of compound **5** on GPVI downstream signalling. As illustrated in Figure 3A and Figure S3, compound **5** (5–10 μM) inhibited the phosphorylation of Fyn, Lyn and p47 protein, a PKC substrate. Figure 3B–D showed the statistical analysis of Fyn, Lyn and p47 protein. Moreover, compound **5** did not affect the PKC activator PDBu‐induced platelet aggregation, suggesting that compound **5** inhibits PKC activity by inhibiting the upstream pathway and not directly inhibiting PKC (Figure 3E). Collectively, these findings indicated that compound **5** can inhibit GPVI signalling.

**FIGURE 4:**
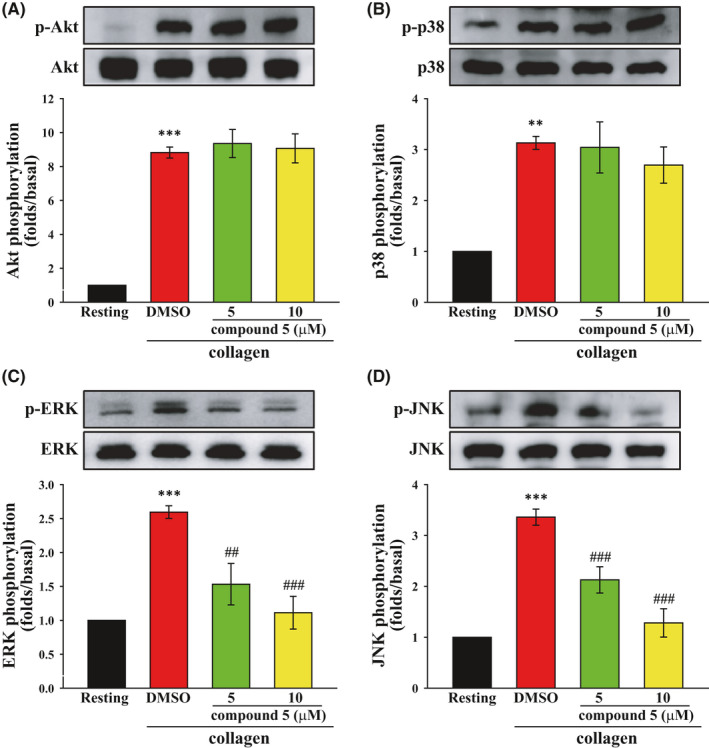
Effects of compound **5** on the collagen‐mediated phosphorylation of Akt, p38 MAPK, ERK and JNK in human platelets. Washed platelets (3.6 × 10^8^ cells/ml) were treated with compound **5** (5 and 10 μM) or DMSO prior to treatment with collagen (1 μg/ml). Protein extracts of platelets were subjected to Western blotting. The total and phosphorylated Akt (A), p38 MAPK (B), ERK (C) and JNK (D) were detected using specific antibodies. Data (A–D) are presented as means ± SEM (*n* = 5). **p < 0.01 and ***p < 0.001 compared with the resting group. ^##^ *p *< 0.01 and ^###^ *p *< 0.001 compared with the DMSO (solvent control) group

Other signalling pathways responsible for collagen‐induced platelet activation were examined. As shown in Figure 4, compound **5** did not affect collagen‐induced Akt or p38 activation but significantly inhibited ERK and JNK activation, suggesting that compound **5** reduced collagen‐induced platelet activation partly through inhibiting the activation of ERK and JNK.

### 3.3 Compound 5 blocked granule release, calcium mobilization and GPIIb/IIIa activation

**FIGURE 5:**
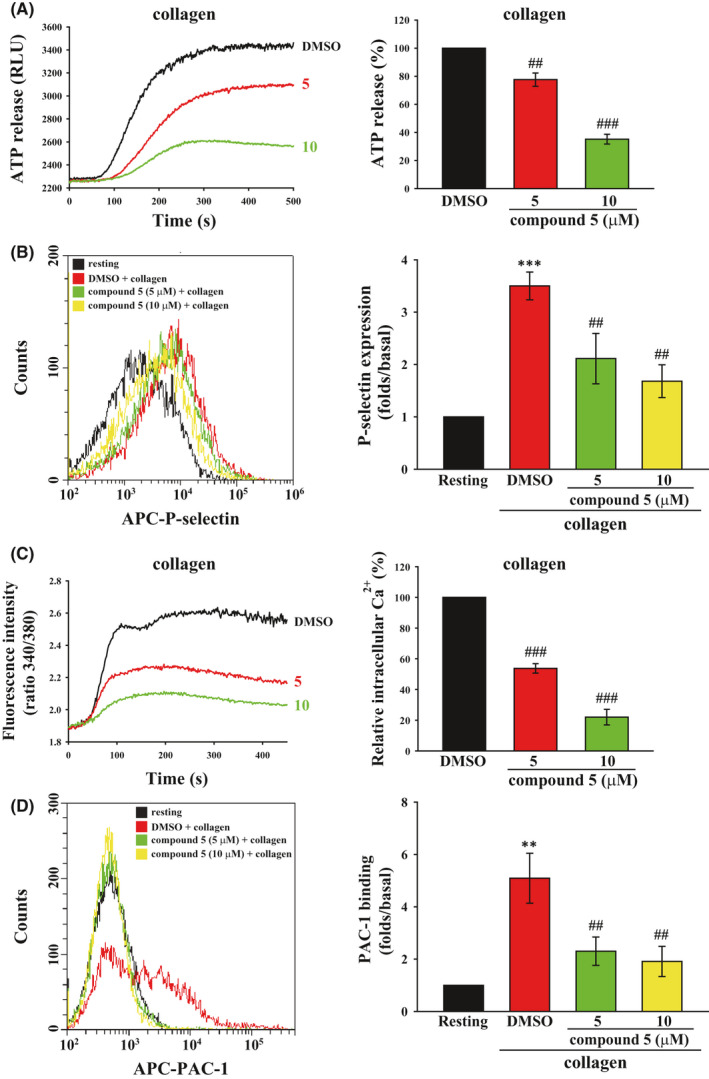
Effects of compound **5** on collagen‐induced granule release, calcium mobilization and GPIIb/IIIa activation in human platelets. Washed platelets (3.6 × 10^8^ cells/ml) were treated with compound **5** (5 and 10 μM) or DMSO and then stimulated with collagen (1 μg/ml) to trigger ATP release (A) and P‐selectin secretion (B), calcium mobilization (C) and GPIIb/IIIa activation (D), which were detected using luciferase/luciferin, APC‐P‐selectin antibodies, Fura 2 and APC‐PAC‐1 antibodies respectively. Data (A‐D) are presented as means ± SEM (*n* = 4). **p < 0.01 and ***p < 0.001 compared with the resting group. ^##^ *p *< 0.01 and ^###^ *p *< 0.001 compared with the DMSO (solvent control) group

Platelet activation signalling can result in substance release from alpha and dense granules, such as ADP and fibrinogen that regulate platelet activation.^25^ Moreover, calcium release can promote granule release. Eventually, the inside‐out signalling can activate GPIIb/IIIa.^3^ Thus, these activation events including granule release, calcium mobilization and GPIIb/IIIa are considered to be indicators of platelet activation. We investigated the effect of compound **5** on these activation events. Granule release was detected by measuring the release of ATP and the surface expression of P‐selectin, which represent the release of dense and alpha granules respectively. As illustrated in Figure 5A,B, collagen markedly induced the release of ATP and the expression of P‐selectin; this release was reversed by compound **5** (5 and 10 μM), indicating that compound **5** can inhibit collagen‐induced granule release. In addition, Fura 2‐AM‐ and APC‐conjugated PAC‐1, which can only recognize the activated form of GPIIb/IIIa,^26^ and Alexa Fluor 647‐conjugated fibrinogen, were used to detect calcium mobilization and GPIIb/IIIa activation respectively. The results revealed that compound **5** (5 and 10 μM) significantly inhibited these two activation events (Figure 5C,D; Figure S4). These findings indicate that compound **5** (5 and 10 μM) could attenuate collagen‐induced platelet activation.

### 3.4 Compound 5 inhibits ex vivo platelet aggregation and pulmonary embolism in mice

**FIGURE 6:**
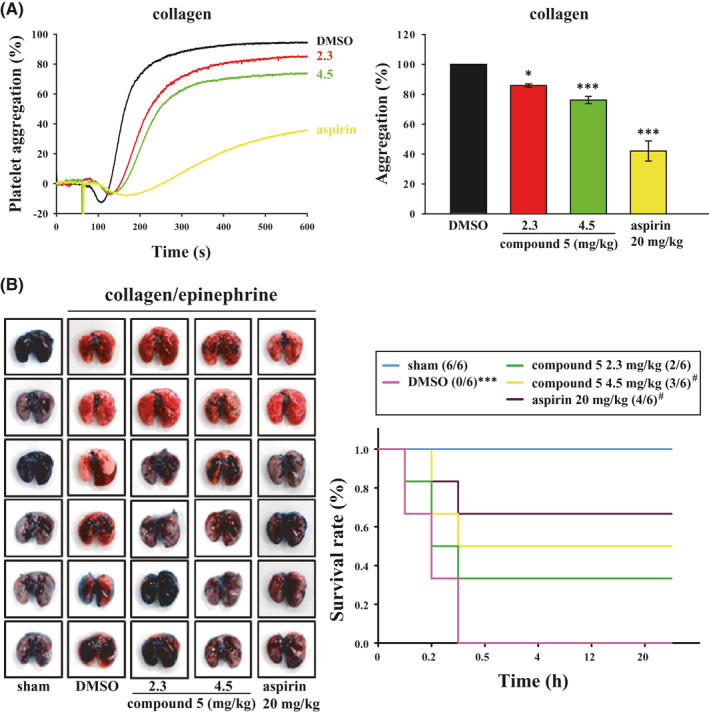
Effects of compound **5** on ex vivo platelet aggregation and in vivo pulmonary embolism in mice. Mice were intravenously administered with various doses of compound **5** (2.3 and 4.5 mg/kg), DMSO (solvent control) or aspirin (20 mg/kg, positive control) for 10 min. Then, (A) whole blood samples were collected, and washed platelets were prepared. Platelet aggregation was recorded 10 min after washed platelets (1.5 × 10^8^ cells/ml) were treated with collagen (1 μg/ml). Data are presented as means ± SEM (*n* = 6). *p < 0.05 and ***p < 0.001 compared with the DMSO (solvent control) group. (B) Mice were injected with collagen/epinephrine to induce pulmonary embolism, which was detected through staining with Evans blue. The survival rate was recorded for 24 h. Data are presented as means ± SEM (*n* = 6). ***p < 0.001 compared with the sham group. ^#^ *p *< 0.05 compared with the DMSO (solvent control) group

Platelets are involved in thrombosis, and antiplatelet agents are commonly used in clinical practice to prevent secondary thromboembolic events.^7^, ^8^ The findings of this study demonstrated that compound **5** exhibited antiplatelet activity in vitro. Thus, we investigated whether compound **5** exerts antithrombotic effects in vivo. To examine platelet aggregation ex vivo, whole blood was obtained from mice after they were injected with compound **5** or aspirin for 10 min. Subsequently, washed mouse platelets were prepared. Platelet aggregation was recorded for 10 min upon collagen treatment. As shown in Figure 6A, compound **5** (2.3 and 4.5 mg/kg) effectively inhibited collagen‐induced platelet aggregation. Moreover, aspirin (20 mg/kg; positive control) exhibited better inhibitory efficacy.

To examine pulmonary embolism in vivo, after mice were injected with compound **5** or aspirin for 10 min, collagen/epinephrine was injected to induce pulmonary embolism, which was observed through staining with Evans blue. The survival rate of mice was evaluated for 24 h. As shown in Figure 6B (left panel), compound **5** (2.3 and 4.5 mg/kg) and aspirin (20 mg/kg) effectively prevented pulmonary thrombosis. Moreover, compound **5** (4.5 mg/kg) and aspirin (20 mg/kg) significantly increased the survival rate by 50% and 67%, respectively, compared with DMSO (Figure 6B, right panel). These results revealed a similar inhibitory tendency of compound **5** and aspirin on ex vivo platelet aggregation and in vivo pulmonary embolism, suggesting that the antithrombotic effect of compound **5** is mediated, at least in part, through its antiplatelet activity.

### 3.5 Compound 5 delayed thrombus formation in mesenteric microvessels in mice

**FIGURE 7:**
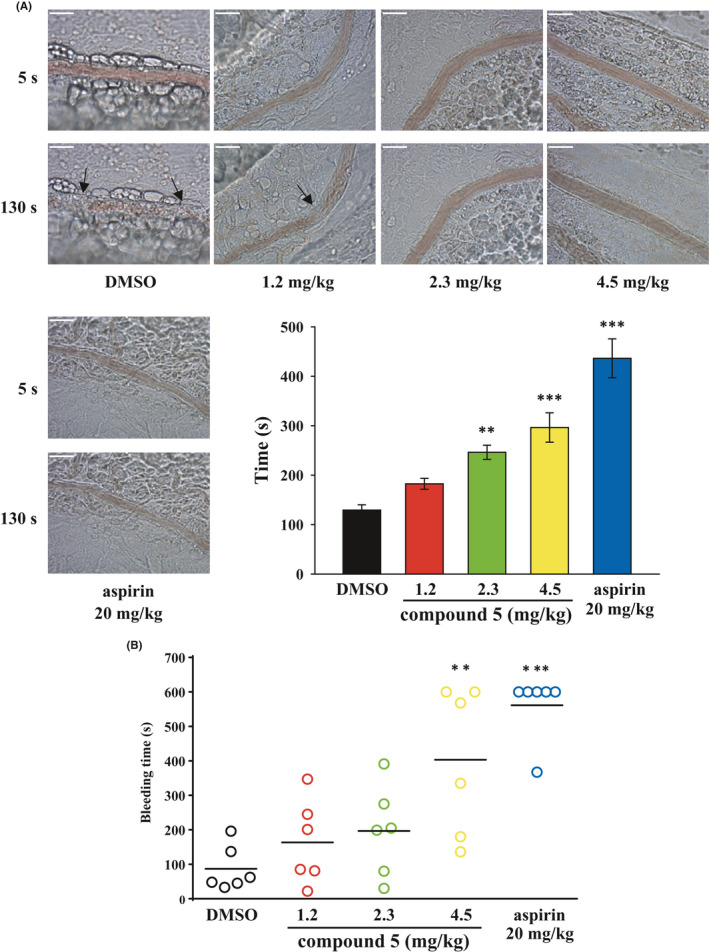
Effects of compound **5** on thrombus formation and haemostasis in mice. Mice were intravenously administered with various doses of compound **5** (1.2, 2.3 and 4.5 mg/kg), DMSO (solvent control) or aspirin (20 mg/kg, positive control) for 10 min. (A) The mesenteric venules (20–30 μm) were irradiated to damage the endothelium, eventually leading to microthrombus formation (arrow). Scale bar = 30 μm. (B) Tail bleeding was induced by cutting the tail, and the bleeding time was recorded until the time at which no sign of bleeding was observed for at least 10 s. Each point in the plot indicates a mouse (*n* = 6). Data (A, B) are presented as means ± SEM (*n* = 6). **p < 0.01 and ***p < 0.001 compared with the DMSO group

We employed another mouse thrombosis model to confirm the antithrombotic effect of compound **5**. In this model, the endothelium was damaged through UV irradiation, resulting in vessel occlusion, which was observed and recorded using a real‐time monitor. As illustrated in Figure 7A, vessel occlusion (arrows) after UV irradiation was observed at approximately 129.3 ± 10.8 s in the DMSO (solvent control)‐treated group, and vessel occlusion was considerably delayed in the aspirin (positive control, 20 mg/kg)‐treated group (approximately 436.4 ± 39.3 s, *p *< 0.001, *n *= 6, compared with the DMSO‐treated group). Moreover, treatment with compound **5** (2.3 and 4.5 mg/kg) significantly prolonged the occlusion time by 117.0 ± 14.4 s and 167.2 ± 29.6 s (*p *< 0.01, *n *= 6; *p *< 0.001, *n *= 6, respectively). These findings suggest that compound **5** exerted an antithrombotic effect. However, new antiplatelet agents must exert strong antithrombotic effects with minimal bleeding risk.^8^ Thus, the mouse tail‐bleeding assay was used to examine whether compound **5** affects haemostasis. As shown in Figure 7B, the aspirin‐treated group exhibited a marked increase in bleeding time compared with the DMSO‐treated group (aspirin, 561.2 ± 38.3 s vs. DMSO, 86.8 ± 26.6 s; *p *< 0.001). Treatment with only a high dose of compound **5** (4.5 mg/kg) significantly affected bleeding time (403.2 ± 87.7 s, *p *< 0.01, *n *= 6) compared with DMSO treatment. However, compound **5** at a low dose (1.2 and 2.3 mg/kg) nonsignificantly increased the risk of bleeding (163.5 ± 49.9 s, *p* = 0.34 and 196.7 ± 53.3 s, *p* = 0.35, respectively). These findings indicate that although compound **5** exerted antithrombotic effects, it tended to cause bleeding.

## 4 DISCUSSION

**FIGURE 8:**
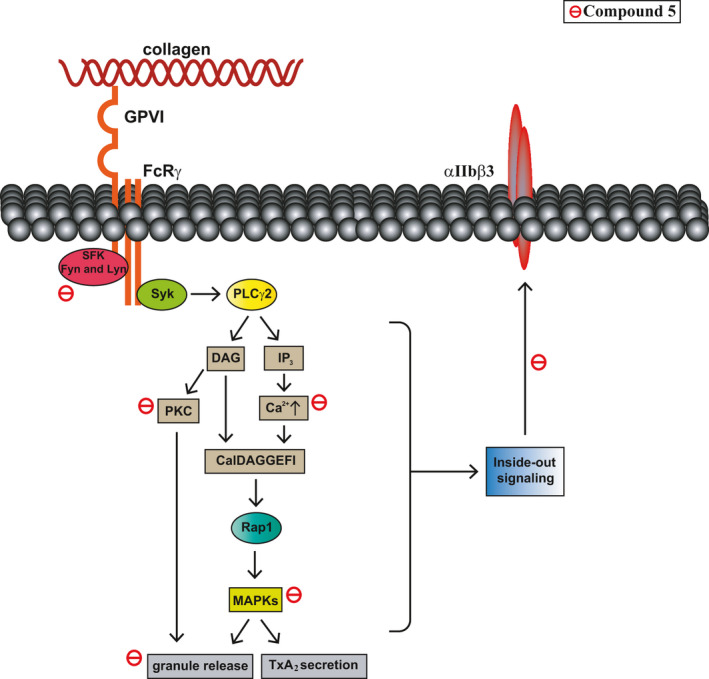
Schematic showing the compound **5**‐mediated inhibition of collagen‐induced platelet activation

This is the first study to demonstrate that compound **5** is more sensitive to the inhibition of collagen‐mediated platelet activation, partly through suppressing GPVI signalling, followed by the inhibition of granule release, calcium mobilization and GPIIb/IIIa activation, eventually blocking platelet activation and thrombus formation (Figure 8). These findings indicate that naphthalimide‐based compounds have antiplatelet and antithrombotic activities. Therefore, compound **5** may serve as a lead compound that can be further modified to synthesize more potent and efficient antiplatelet agents with minimal bleeding risk.

Naphthalimides have been used as a core scaffold for the development of antitumour and anti‐inflammatory agents.^9^, ^10^ They can intercalate with DNA and inhibit topo II due to their planar and heteroaromatic structure. Thus, many new naphthalimide derivatives have been developed as anticancer agents.^9^, ^10^ We previously synthesized several naphthalimide derivatives and found them to exhibit cytotoxic effects on B16F10 melanoma cells and reduce lung metastasis at least partly through the inhibition of topo II activity.^15^, ^16^ Among the naphthalimide derivatives we synthesized, compound **5** was found to exert the strongest inhibitory effect on topo II activity.^16^ At a concentration of 10 μM, compound **5** almost completely inhibited topo II activity. In the present study, we found that compound **5** (10 μM) blocked platelet aggregation and platelet activation events including granule release, calcium mobilization and GPIIb/IIIa activation. This finding implies that naphthalimides can serve as a core scaffold for antiplatelet agents. In addition, compound **5** was observed to be more sensitive to collagen‐induced platelet activation compared with thrombin‐ and U46619‐induced platelet activation, indicating its action on the collagen receptor downstream signalling pathway, such as Fyn, Lyn and PKC.

Platelets play a crucial role in arterial thrombosis that can cause heart attack and stroke. Atherosclerosis is the leading cause of heart attack and stroke. In addition to platelet activation, several risk factors such as lipid accumulation, endothelial cell activation, vascular smooth muscle cell proliferation and inflammation^27^ are involved in atherosclerosis. Previously, **7b**, a naphthalimide derivative, was reported to inhibit inflammation through suppressing NF‐κB activation in RAW264.7 macrophages.^17^ In addition, a novel naphthalimide (NAP‐6) could selectively target breast cancer cells by activating the arylhydrocarbon receptor (AhR) pathway.^28^ Although the role of AhR remains controversial between pro‐ and anti‐inflammatory responses in atherosclerosis,^29^, ^30^ AhR was recently reported to play a protective role in promoting the maintenance of lesion cap integrity and reducing the transition of smooth muscle cells to chondromyocytes during atherosclerosis.^31^ Together, these observations suggest that naphthalimide derivatives exert anti‐inflammatory effects to prevent cardiovascular diseases such as atherosclerosis and stroke. However, whether naphthalimide‐based compound **5** exerts anti‐inflammatory effect remains to be determined.

Akt is essential for collagen‐induced platelet aggregation,^32^ and an SFK inhibitor was reported to completely inhibit GPVI‐induced Akt phosphorylation.^6^ Fyn‐deficient platelets exhibited decreased Akt phosphorylation induced by convulxin.^6^ Taken together, these findings suggest that SFK activates Akt in GPVI signalling. However, compound **5** inhibited the collagen‐mediated phosphorylation of SFK (Lyn and Fyn) but did not affect the collagen‐mediated phosphorylation of Akt. This discrepancy should be examined in future studies.

Platelets have MAPKs that mainly consist of three families: ERKs, JNKs and p38/SAPKs.^4^ MAPKs are activated by platelet agonists through the activation of their receptors and can regulate platelet activation and thrombus formation.^4^ ERK1/2 may regulate cPLA_2_ phosphorylation and AA metabolism into TxA_2_, subsequently causing dense granule release.^4^ JNK1 regulates dense granule release by affecting TxA_2_ generation.^4^ p38 regulates cPLA_2_ phosphorylation and activity as well as α‐granule and dense granule secretion.^4^ MAPKs can support clot retraction and GPIIb/IIIa activation.^4^, ^33^, ^34^, ^35^ Moreover, pharmacological inhibition or gene deletion of MAPKs significantly prolonged thrombus formation.^4^ In the present study, compound **5** inhibited granule release, GPIIb/IIIa activation and thrombus formation, in part through suppressing ERK and JNK phosphorylation.

Targeting the collagen receptor GPVI can be a favourable strategy for preventing thrombosis while preserving haemostasis.^8^ Patients with GPVI deficiency only exhibited mild bleeding. Moreover, GPVI‐deficient mice exhibited protection against arterial thrombosis.^8^ In clinical trials, two classes of drugs, a humanized anti‐GPVI Fab fragment (ACT01) and a dimeric GPVI‐Fc fusion protein (Revacept), could inhibit the interaction between platelets and collagen but did not affect general haemostasis.^36^, ^37^ In addition, studies have reported that fibrin and fibrinogen may activate GPVI and stabilize thrombi.^38^, ^39^, ^40^ These findings indicate that targeting GPVI may be a promising antithrombotic therapy. However, our in vivo study findings indicated that compound **5** could effectively prevent thrombus formation but showed a tendency to cause bleeding, especially at a high dose; this implies that compound **5** may affect other targets, such as the coagulation cascade, and the implication requires elucidation through further research. Therefore, although compound **5** may act as a lead compound for the design of new naphthalimide‐based antiplatelet agents, the bleeding side effect must be eliminated or minimized during future drug development.

Glycoprotein VI was recently reported to promote metastasis by interacting with cancer cell‐derived galectin‐3.^41^ Platelet activation causing the formation of aggregates on the surface of circulating tumour cells may protect against immune cell attack.^42^ Previously, we demonstrated that naphthalimide derivatives could prevent lung metastasis of melanoma cells through the inhibition of topo II.^15^, ^16^ Our present data revealed that a naphthalimide derivative (compound **5**) could prevent platelet activation, likely through the inhibition of GPVI signalling. In addition to the inhibition of topo II activity, whether the antimetastatic effect of naphthalimide derivatives can be attributed to their ability to inhibit platelet activation should be examined in future studies.

In conclusion, our findings indicated that the naphthalimide derivative compound **5** could exert antiplatelet and antithrombotic effects, at least in part, through the suppression of GPVI signalling. This naphthalimide derivative can serve as a core scaffold for developing novel antiplatelet agents to treat patients with cardiovascular diseases if the potential adverse effect of bleeding is eliminated or minimized.

## CONFLICT OF INTEREST

The authors declare that there is no conflict of interest.

## AUTHOR CONTRIBUTION

**Tzenge‐Lien Shih:** Formal analysis (equal); Investigation (equal); Resources (lead); Writing‐review & editing (equal). **Kuan‐Hung Lin:** Conceptualization (lead); Funding acquisition (lead); Methodology (equal); Validation (equal); Writing‐original draft (lead). **Ray‐Jade Chen:** Funding acquisition (lead); Writing‐review & editing (equal). **Ting‐Yu Chen:** Data curation (equal); Formal analysis (equal); Investigation (equal). **Wei‐Ting Kao:** Formal analysis (equal); Resources (equal). **Jen‐Wei Liu:** Formal analysis (equal); Resources (equal). **Hsueh‐Hsiao Wang:** Writing‐review & editing (equal). **Hsien‐Yu Peng:** Writing‐review & editing (equal). **Yu‐Yo Sun:** Writing‐review & editing (equal). **Wan‐Jung Lu:** Conceptualization (lead); Data curation (equal); Formal analysis (equal); Funding acquisition (lead); Validation (equal); Writing‐original draft (lead); Writing‐review & editing (lead).

## Supporting information

Figure S1‐S4

## Data Availability

The data that support the findings of this study are available from the corresponding author upon reasonable request. Some data may not be made available because of privacy or ethical restrictions.
