# Supplementary material for: A novel naphthalimide derivative reduces platelet activation and thrombus formation via suppressing GPVI
Source: J Cell Mol Med. 2021 Aug 27;25(19):9434–46. doi: 10.1111/jcmm.16886 (PMC8500964; doi:10.1111/jcmm.16886)
Supplement: Supplementary file 1 — Figure S1‐S4 [file JCMM-25-9434-s001.docx]

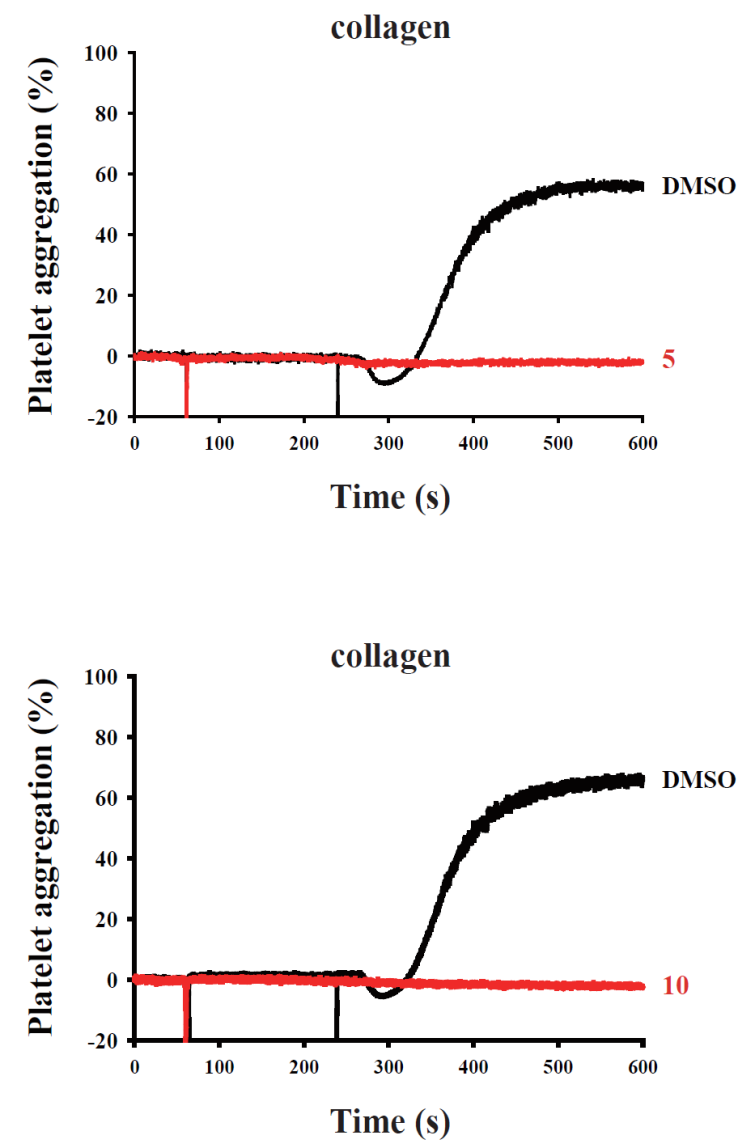


**supplementAry Figure 1** Effects of compound **5** on platelet shape change. Shape change was observed for 10 min after washed platelets (3.6 × 10^8^ cells/mL) were treated with compound **5** (5 and 10 μM).


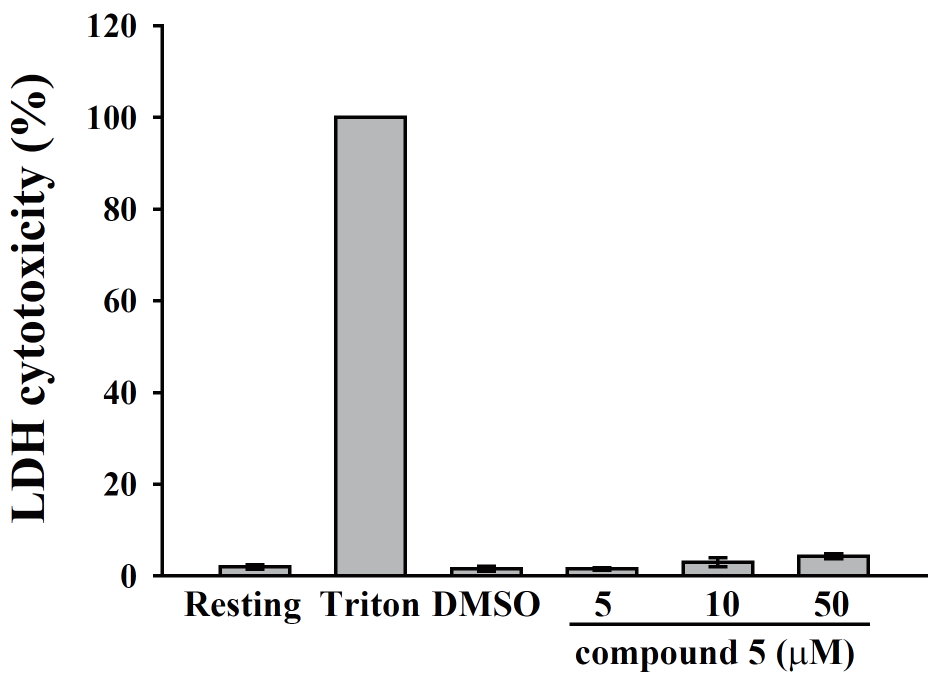


**supplementAry Figure 2** Effects of compound **5** on lactate dehydrogenase (LDH) release. The washed platelets (3.6 × 10^8^ cells/mL) were incubated with Tyrode’s solution (resting), DMSO (solvent control), or various concentrations of compound **5** (5–50 μM) for 10 min at 37°C, and the supernatant was collected to measure LDH release by using the LDH assay kit. LDH activity was expressed as the percentage of total enzyme activity, which was measured as platelets lysed with 0.5% Triton X-100.

**
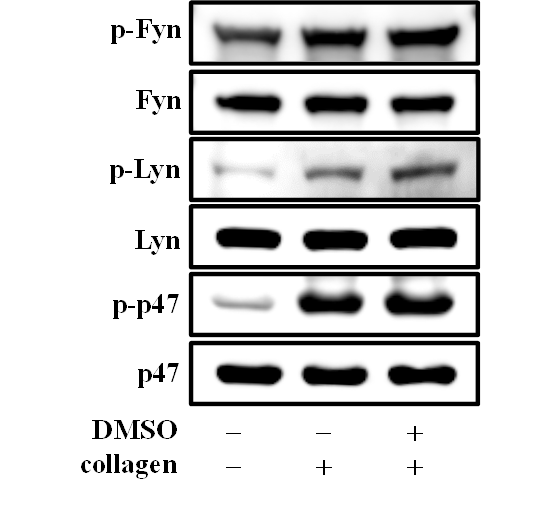
**

**supplementAry Figure 3** Collagen-mediated phosphorylation of Fyn, Lyn, and PKC substrates in human platelets. Washed platelets (3.6 × 10^8^ cells/mL) were treated with or without DMSO prior to treatment with collagen (1 μg/mL). Protein extracts of platelets were subjected to Western blotting. The total and phosphorylated Fyn, Lyn, and PKC substrates (p47) were detected using specific antibodies. Profiles are the representative examples of three similar experiments.


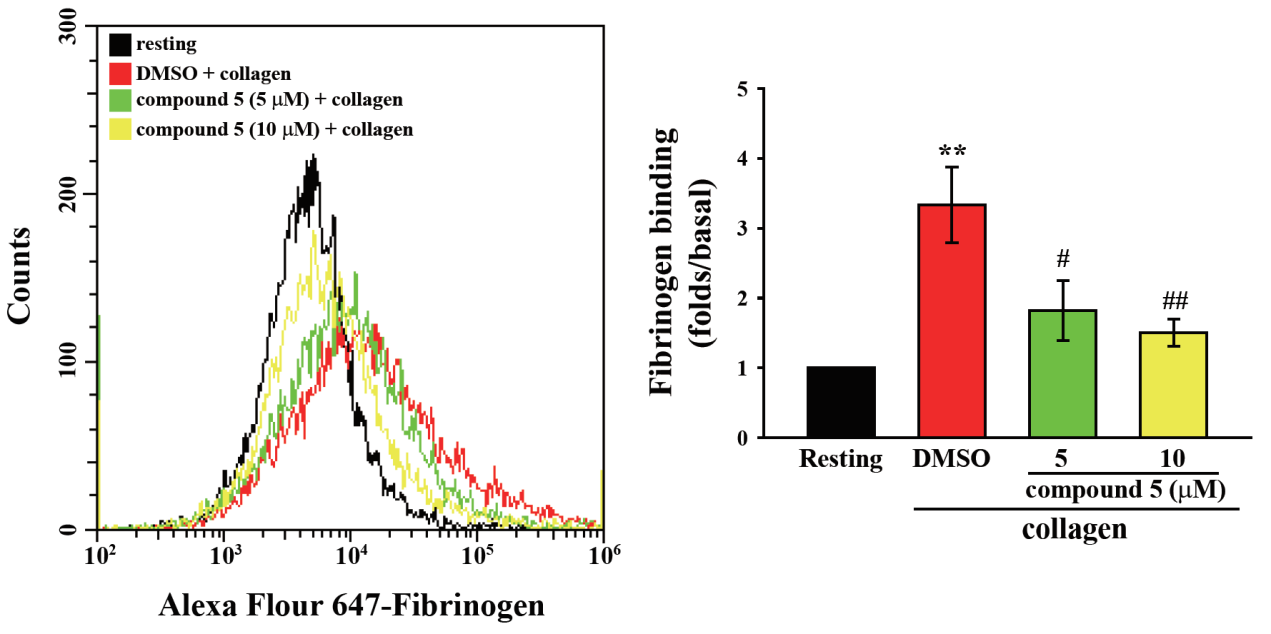


**supplementAry Figure 4** Effects of compound **5** on collagen-induced granule GPIIb/IIIa activation in human platelets. Washed platelets (3.6 × 10^8^ cells/mL) were treated with compound **5** (5 and 10 μM) or DMSO and then stimulated with collagen (1 μg/mL) to trigger GPIIb/IIIa activation, which was detected using Alexa Fluor 647-conjugated fibrinogen. Data are presented as means ± SEM (*n* = 4). ***P <* .01 and ****P <* .001 compared with the resting group. ^##^*P <* .01 and ^###^*P <* .001 compared with the DMSO (solvent control) group.
